# Efficacy and Safety of Sotrovimab Versus Oral Antiviral for Early Treatment in High-Risk Patients in Omicron Era: A Multicenter Retrospective Study

**DOI:** 10.3390/pathogens14030216

**Published:** 2025-02-22

**Authors:** Antonio Russo, Mariantonietta Pisaturo, Chiara Cacace, Augusta Troise, Gabriele Granata, Pierantonio Grimaldi, Enrico Allegorico, Francesca Ambrisi, Martina Papillo, Fabio Giuliano Numis, Nicola Coppola

**Affiliations:** 1 Infectious Diseases, Department of Mental Health and Public Medicine, University of Campania “L. Vanvitelli”, 80131 Napoli, Italy; antoniorusso.ar.ar@gmail.com (A.R.); mariantonmietta.pisaturo@unicampania.it (M.P.); ccacace56@gmail.com (C.C.); gabriele.granata@studenti.unicampania.it (G.G.); pierantonio.grimaldi@studenti.unicampania.it (P.G.); francescambrisi@gmail.com (F.A.); 2 Emergency Unit, PO Santa Maria Delle Grazie, 80078 Pozzuoli, Italy; augusta.troise@aslnapoli2nord.it (A.T.); enrico.allegorico@aslnapoli2nord.it (E.A.); martina.papillo@aslnapoli2nord.it (M.P.); fabiogiuliano.numis@aslnapoli2nord.it (F.G.N.)

**Keywords:** early therapy, sotrovimab, safety, Omicron variant, COVID-19, SARS-CoV-2 infection

## Abstract

Introduction: High-risk patients with COVID-19 benefit from early treatment to prevent severe outcomes. Sotrovimab, a monoclonal antibody, and oral antivirals such as nirmatrelvir/ritonavir and molnupiravir have been used for early intervention, but their comparative efficacy and safety, particularly during the Omicron-dominant phase, require further evaluation. Methods: A multicenter, retrospective study performed in southern Italy including all adult patients who received early antiviral treatment (sotrovimab or nirmatrelvir/r or molnupiravir) between January 2022 and February 2024 (omicron phase). Demographic, clinical, and treatment-related data were analyzed to assess primary endpoints of 28-day mortality and hospitalization. Logistic regression models identified predictors of key outcomes. Results: A total of 668 high-risk patients treated with sotrovimab (n = 326) or oral antivirals (n = 342: 69 with molnupiravir and 273 with nirmatrelvir/ritonavir) were included. There was no significant difference in 28-day mortality between groups (0.8% sotrovimab vs. 1.8% oral antivirals; *p* = 0.679). However, patients treated with sotrovimab exhibited a longer median time to SARS-CoV-2 negativization (13 vs. 11 days; *p* = 0.008) and higher non–COVID-19-related hospitalizations (2.45% vs. 0%; *p* = 0.003). Multivariable analysis identified cardiovascular or cerebrovascular diseases as the sole significant predictor of prolonged viral positivity (OR 1.585, 95% CI 1.072–2.345; *p* = 0.021). Additionally, immunocompromised status (OR 16.929, 95% CI 1.835–156.170; *p* = 0.013) and chronic non-COVID-19 oxygen therapy (OR 10.714, 95% CI 1.623–70.725; *p* = 0.014) were strongly associated with mortality. Conclusions: Sotrovimab and oral antivirals demonstrated similar efficacy in preventing mortality and hospitalization among high-risk patients. Patient-specific factors, particularly cardiovascular comorbidities and immunosuppression, significantly influenced outcomes and should guide treatment choices.

## 1. Introduction

Despite the rapid development and deployment of effective vaccines, the persistence of viral transmission and the emergence of new variants underscore the need for effective therapeutic options, particularly in the early stages of infection and in patients with immune depression to prevent the disease progression [1].

As highlighted by many studies, the presence of comorbidities can predict the progression of COVID-19 in terms of hospitalization, oxygen therapy, and death [2,3,4,5]. Early intervention with antiviral agents can significantly reduce the risk of disease progression, hospitalization, and mortality, especially among high-risk populations [6,7,8,9,10]. Oral antivirals such as nirmatrelvir/ritonavir and molnupiravir have received emergency use authorization for treating mild-to-moderate COVID-19 in patients at high risk of developing severe illness [11,12]. In addition to oral antivirals, one of the first authorized antivirals was remdesivir, initially used in patients with severe illness and later as an early treatment. It is currently still used both in hospitalized patients and as early treatment, administered intravenously, and remains effective despite viral mutations. [13,14,15] Sotrovimab, a monoclonal antibody targeting a conserved epitope of the SARS-CoV-2 spike protein, was initially authorized for emergency use and demonstrated efficacy in neutralizing a broad range of variants [16,17,18]. Its mechanism of action involves binding to the spike protein, thereby blocking viral entry into host cells [19]. However, with the emergence of new variants, particularly the Omicron subvariants, concerns arose regarding its effectiveness [20] and indicated that sotrovimab’s neutralizing activity was significantly reduced against certain Omicron sublineages [21,22]. As a result, regulatory agencies in both Italy and the United States have suspended the authorization of sotrovimab [23,24]. Recently, studies investigating the clinical evolution of patients treated with sotrovimab as early treatment highlighted its efficacy in patients with mild-to-moderate SARS-CoV-2 compared to treatment with or without other early treatment strategies [25,26]. Considering the rapid evolution of SARS-CoV-2 variants, the continuous assessment of therapeutic efficacy is needed to improve clinical outcomes especially in the high-risk population.

This study aims to evaluate, in a real-world experience, the efficacy of sotrovimab in Omicron subvariants compared to the oral antivirals nirmatrelvir and molnupiravir in the early treatment of COVID-19 in a high-risk population, considering mortality and hospitalization for COVID-19 at 28 days from the first dose of early treatment. The findings are expected to inform therapeutic decision-making and contribute to improving the outcomes of at-high-risk-of-progression patients with SARS-CoV-2 infection.

## 2. Materials and Methods

### 2.1. Study Design and Setting

We performed a two-center, observational, retrospective study involving two units in two cities in the Campania region in southern Italy: Naples and Pozzuoli. All adult (≥18 years) patients at high risk of progression, who performed early treatment for COVID-19, with a diagnosis of SARS-CoV-2 infection from 3 January 2022 to 28 February 2024 in one of the two centers participating in this study were included. We decided to include patients from 3 January 2022, considering the Italian regional report [27] that was the first report which defined a prevalence of more than 50% (80.75%) of the Omicron variant.

The early treatments prescribed were those indicated by the regulatory bodies Agenzia Nazionale del Farmaco (AIFA) and the European Medicine Agency (EMA) [28], and the drug was chosen based on the indications from regulatory bodies [29,30,31] and clinical decisions.

From the cohort, we included all patients who were treated with nirmatrelvir/ritonavir, molnupiravir, or sotrovimab. All patients who performed oral antiviral treatment or monoclonal antibody treatment were followed up for 28 days through telematic or on-site evaluation. Oral antivirals (molnupiravir or nirmatrelvir/ritonavir) were prescribed in the hospital, and the patients performed the treatment at home; patients who performed monoclonal antibody (sotrovimab) performed the single dose in the hospital and then were discharged at home.

All demographic and clinical data of patients with SARS-CoV2 infection enrolled in the cohort were collected in an electronic database. From this database, we extrapolated the data for the present study.

This study was approved by the Ethics Committee of the University of Campania L. Vanvitelli, Naples (n°10877/2020). All procedures performed in this study were in accordance with the ethics standards of the institutional and/or national research committee and with the 1964 Helsinki declaration and its later amendments or comparable ethics standards. Informed consent was obtained from all participants included in this study.

### 2.2. Statistical Analysis

For the descriptive analysis, categorical variables were presented as absolute numbers and their relative frequencies. Continuous variables were summarized as the median and interquartile range (Q1–Q3). We performed a comparison of patients who performed treatment with sotrovimab or one oral antiviral and sub-analysis with Pearson chi-square or Fisher’s exact test for categorical variables and Student’s *t*-tests or Mann–Whitney tests for continuous variables. To evaluate the distribution of continuous variables, we used the Shapiro–Wilk test. To assess the association between clinical variables and the outcome (time of negativization less or more of 12 days; death/no death during follow up), a binary logistic regression was used. In the first stage, a univariate analysis was performed, including in the multivariable model all variables with a *p*-value below 0.05 and/or those identified a priori as clinically relevant. The final selection of variables was carried out using a stepwise approach, retaining only predictors independently associated with the outcome. The estimated coefficients were expressed as Odds Ratios (ORs) with corresponding 95% confidence intervals. A *p* value less than 0.05 was set for statistical significance. Analyses were performed by STATA 16 [32].

## 3. Results

During the study period, 668 patients with diagnosis of SARS-CoV-2 infection and mild-to-moderate symptoms treated with nirmatrelvir/ritonavir, molnupiravir, or sotrovimab were included in this study: specifically, 326 patients were treated with sotrovimab and 342 with nirmatrelvir/ritonavir or molnupiravir (molnupiravir in 69 patients and nirmatrelvir/ritonavir in 273 patients, Table 1 and Table 2, Supplementary Tables S1–S4).

Table 3 shows the demographic and clinical characteristics of patients included in the study grouped by the different early treatment performed (sotrovimab vs. oral treatment). There were no significant differences between the two groups considering the gender (male 47.2% vs. 50.9%, *p* = 0.354) and age (median 70 vs. 69, *p* = 0.283). Patients who performed at least one vaccination for SARS-CoV-2 were higher in the oral antiviral group (91.4% vs. 75%, *p* ≤ 0.001) as well as the percentage of patients who performed all vaccine doses advised (79% vs. 55.4%, *p* ≤ 0.001). Considering clinical characteristic at admission, the patients with oncological or hematological disease were higher in the oral antiviral group (18.1% vs. 10.7%, *p* = 0.008); on the other hand, a higher rate of patients with cardiovascular disease (48.8% vs. 34.2%, *p* = 0.001), decompensated diabetes mellitus (17.5% vs. 9.9, *p* = 0.005), and neurological disease (8.3% and 3.3%, *p* = 0.003) were found in the sotrovimab group. The patients with more than 65 years old were more frequently treated with sotrovimab (51.5% vs. 38.9%, *p* = 0.001).

Table 4 shows the outcome of patients included in this study. Between the two groups of treatment, no difference was observed in the prevalence of patients who regularly finish the treatment and of those who stop the drug for adverse events, clinical decisions, hospitalization, or for patients’ decisions (*p* = 0.433). Moreover, no difference was found in the percentage of patients dead within 28 days after the start of treatment (0.8% vs. 1.8%, *p* = 0.679). The five patients who died, died from causes unrelated to the SARS-CoV-2 infection. We found statistical difference in the median time for negativization of SARS-CoV-2 swap, revealing longer positivity in sotrovimab patients (13 days vs. 11 days, *p* = 0.008) (Table 4, Supplementary Table S7) and when considering the hospitalization for reasons other than COVID-19 within 28 days from the start of antiviral treatment there was a higher rate in the sotrovimab group (2.45% vs. 0%, *p* = 0.003) (Table 4, Supplementary Tables S5 and S6). Table 2 showed the data considering separately each oral antiviral: the patients treated with sotrovimab compared with nirmatrelvir/ritonavir showed a higher time to negativization (13 days vs. 11 days, *p* = 0.002) and a higher rate of hospitalization for reasons other than COVID-19 (2.5% vs. 0%, *p* = 0.007) (Table 2).

Considering the statistical difference in the median time of negativization of swab for SARS-CoV-2, we decided to define two groups. The median time of negativization was 12 days; so, the first group included all patients with a time to negativization less than or equal to 12 days (244 patients), and the second group included all patients with more than 12 days (215 patients; Table 5). Patients with a time to negativization greater than 12 days showed a higher time elapsed from symptom onset to 1st drug dose (3 days vs. 2 days, *p* = 0.045), a lower rate of patients who received all advised doses of vaccine (67.1% vs. 77.6%, *p* = 0.013), and a higher percentage of patients with cardiac or cerebrovascular disease (45.6% vs. 33.6%, *p* = 0.009) (Table 5). In addition, patients treated with nirmatrelvir/r appeared to have a time of negativization less than 12 days more frequently compared to sotrovimab alone or compared to sotrovimab and molnupiravir (Table 5).

The multivariable analysis aiming to assess the risk factors for a time to negativization greater than 12 days showed that previous cardiac or cerebrovascular disease is the only risk factor for a time to negativization greater than 12 days (OR 1.585, 95% CI 1.072–2.345; *p* = 0.021) (Table 6).

The five (0.74%) patients dead during follow up had a more frequent history of chronic non-COVID-19-related O_2_ therapy (40% vs. 6.3%, *p* = 0.037), lower oxygen saturation in the blood with a finger sensor before early therapy (92% vs. 96%, *p* = 0.005), and were more likely to receive molnupiravir and sotrovimab rather than nirmatrelvir/r (Table 7). The multivariable analysis, including clinically relevant factors, such as immunocompromised condition, patient in chronic non-COVID-19 O_2_ therapy, and the early antiviral administered (molnupiravir or nirmatrelvir/r or sotrovimab), showed that immunocompromised patients (OR 16.929, 95% CI 1.835–156.170) and chronic O_2_ therapy not related to COVID-19 (OR 10.714, 95% CI 1.623–70.725) were the two variables linked to death during follow up in this population (Table 8).

## 4. Discussion

This multicenter, observational, retrospective study evaluated the effectiveness of early treatment with sotrovimab compared to oral antivirals, nirmatrelvir/ritonavir and molnupiravir, in patients with mild-to-moderate COVID-19 during a period dominated by the Omicron variant in southern Italy. Our findings contribute to the growing body of evidence assessing therapeutic options amid evolving SARS-CoV-2 variants. The primary outcome demonstrated no significant difference in mortality rates within 28 days between the sotrovimab group and the oral antiviral group (0.8% vs. 1.8%, *p* = 0.679). Additionally, there were no significant differences in treatment completion rates or discontinuations due to adverse events between the two groups (*p* = 0.433). These findings are consistent with previous studies, including our meta-analysis [25], which found no significant difference in mortality or hospitalization rates between sotrovimab and other early COVID-19 therapies targeting Omicron subvariants. It is worth noting, however, that some studies have reported that Nirmatrelvir exhibits superior efficacy in preventing hospitalizations and deaths [33]. However, interestingly, the patients treated with sotrovimab exhibited a longer median time to viral negativization compared to those receiving oral antivirals (13 days vs. 11 days, *p* = 0.008). The sub-analysis performed to evaluate the determinant of longer time to negativization highlighted that the most important factor increasing this outcome was the presence at baseline of cerebrovascular or cardiovascular disease (including heart failure, coronary disease, cardiomyopathy, arterial hypertension with related organ damage, and stroke), that probably led to the clinical decision to perform certain early treatment strategies with less drug interaction.

Another notable finding was the higher rate of non-COVID-19-related hospitalizations within 28 days in the sotrovimab group compared to the oral antiviral group (2.45% vs. 0%, *p* = 0.003). This evidence is nevertheless counterintuitive considering the safety of monoclonal antibodies compared to oral antivirals, despite what has been reported in other studies [33]. This disparity may reflect the higher prevalence of comorbid conditions such as cardiovascular disease, decompensated diabetes mellitus, and neurological disorders in patients receiving sotrovimab, but six of eight patients hospitalized for non-COVID-19 disease were admitted due to adverse reactions during the administration of sotrovimab, which, if present, more often require at least short-term observation, if not hospitalization, compared to their oral counterparts. The vaccination status differed significantly between the two groups, with a higher percentage of vaccinated individuals in the oral antiviral group (91.4% vs. 75%, *p* ≤ 0.001) and more patients having received all recommended vaccine doses (79% vs. 55.4%, *p* ≤ 0.001). Vaccination is known to reduce disease severity and may have influenced the selection of treatment modality, with clinicians perhaps opting for oral antivirals in vaccinated patients due to anticipated milder disease progression.

Our study has several limitations that should be acknowledged. The sample size of patients is lower compared to the larger national and international cohorts [26,34]. The retrospective design may introduce selection bias, and the allocation of treatments was based on clinical decisions rather than randomization, potentially affecting the comparability of the groups; moreover, the number of patients treated with molnupiravir was scarce since after a short time from this study’s start it was no longer possible to prescribe it. Moreover, according to the study design, patients who refused the treatment despite having an indication were not included, and, therefore, there is no control group of patients who received no treatment. The study period was limited to the dominance of the Omicron variant, and our findings may not be generalizable to other variants or future strains with different susceptibilities to these therapies. Additionally, the lack of data on the specific Omicron sublineages limits our ability to assess the efficacy of treatments against subvariants. Despite these limitations, our study provides valuable real-world evidence on the effectiveness of sotrovimab and oral antivirals during a critical phase of the pandemic. The comparable mortality and hospitalization rates suggest that both treatment options remain viable for managing mild-to-moderate COVID-19 in high-risk patients.

An important consideration when selecting nirmatrelvir/ritonavir is its potential for drug–drug interactions, which often limits its use in patients receiving medications for chronic conditions, such as cardiovascular drugs, immunosuppressants, and certain psychiatric medications [35]. Nirmatrelvir is co-administered with ritonavir, a potent inhibitor of cytochrome P450 enzymes, particularly CYP3A4, leading to significant interactions that may necessitate dose adjustments or even discontinuation of concomitant therapies [35]. This challenge often compels clinicians to choose alternatives like molnupiravir or sotrovimab, both of which have much fewer interactions and are better tolerated in patients with polypharmacy [35]. Nevertheless, it should be noted that there remains the possibility of the emergence of a variant that may not respond to monoclonal antibodies. As of now, nirmatrelvir/ritonavir remains the only oral antiviral option in Italy, with molnupiravir no longer available [36]. Remdesivir, another antiviral option, is available but requires intravenous administration over three consecutive days, which may reduce patient adherence to early treatment due to logistical challenges, especially in non-hospitalized settings [37], but the efficacy is to date debated [38].

In conclusion, our study indicates that early treatment with either sotrovimab or oral antivirals is effective in preventing disease progression in patients with mild-to-moderate COVID-19 during the Omicron-dominated period. Future research should focus on prospective studies with randomized controlled designs to validate these findings and explore the impact of emerging variants on treatment efficacy.

## Figures and Tables

**Table 1:** Demographic and clinical data of patients included in this study grouped by the early treatment performed.

	**All Patients**	**Molnupiravir**	**Nirmatrelvir/r**	**Sotrovimab**
**Patients included**	**668**	**69 (10.33%)**	**273 (40.87%)**	**326 (48.80%)**
**Males, N° (%)**	**238 (49.1)**	**35 (50.7)**	**139 (50.9)**	**154 (47.2)**
**Age in years, median (Q1–Q3)**	**69 (58–79)**	**63 (57–78)**	**70 (59–78)**	**70 (57–80)**
**Time from symptom onset to 1st drug dose, median (Q1–Q3)**	**2 (1–4)**	**3 (2–4)**	**2 (1–3)**	**3 (2–6)**
**N° (%) anti-SARS CoV-2 vaccinated patients**	**550 (83.5)**	**55 (80.9)**	**255 (94.1)**	**240 (75)**
**N° (%) patients who received all advised doses according to the vaccine**	**439 (67.3)**	**50 (73.5)**	**210 (80.5)**	**179 (55.4)**
**N° (%) patients in chronic non COVID-19 O_2_ therapy**	**44 (6.6)**	**8 (11.6)**	**9 (3.3)**	**27 (8.3)**
**N° (%) oncologic/hematologic active disease**	**97 (14.5)**	**10 (14.5)**	**52 (19)**	**35 (10.7)**
**N° (%) CKD patients**	**58 (9.7)**	**2 (2.9)**	**21 (7.7)**	**35 (10.7)**
**N° (%) COPD or other chronic disease patients**	**92 (13.8)**	**9 (13)**	**39 (14.3)**	**44 (13.5)**
**N° (%) immunocompromised, innate or acquired, patients**	**137 (20.5)**	**20 (29)**	**46 (16.8)**	**71 (21.8)**
**N° (%) obese patients**	**119 (17.8)**	**18 (26.1)**	**40 (14.7)**	**61 (18.7)**
**N° (%) cardiac/cerebrovascular disease patients (HF, coronary disease, cardiomyopathy, HT with related organ damage, stroke)**	**276 (41.3)**	**38 (55.1)**	**79 (28.9)**	**159 (48.8)**
**N° (%) decompensated diabetes mellitus patients (Hb1A** **≥ 9.0% or 75 *mol*/*mol*) or chronic complications**	**91 (13.6)**	**14 (20.3)**	**20 (7.3)**	**57 (17.5)**
**N° (%) over 65 y/o patients**	**301 (45.1)**	**26 (37.7)**	**107 (39.2)**	**168 (51.5)**
**N° (%) liver chronic disease**	**13 (1.9)**	**1 (1.4)**	**2 (0.7)**	**10 (3.1)**
**N° (%) neurological disease**	**37 (5.5)**	**2 (2.9)**	**8 (2.9)**	**27 (8.3)**
**SpO_2_ at admission, Median (Q1–Q3)**	**96 (95–98)**	**97 (95–98)**	**96 (95–97)**	**96 (95–98)**

Abbreviations and acronyms: SARS CoV-2: Severe Acute Respiratory Syndrome Corona Virus-2; COVID-19: Corona Virus Disease-2019; CKD: Chronic Kidney Disease; COPD: Chronic Obstructive Pulmonary Disease; HF: Heart Failure; HT: Hyper Tension; Hb1A: Hemoglobin 1A; y/o: Years old.

**Table 2:** Outcome of patients included in this study grouped by the early treatment performed.

	All Patients Included	Molnupiravir	Nirmatrelvir/r	Sotrovimab	Sotrovimab vs. Nirmatrelvir/r *p* Value	Sotrovimab vs. Molnupiravir *p* Value
**N° (%) of patients who finished the treatment**	593 (88.8)	67 (97.1)	243 (89)	283 (86.8)	0.387	**0.028**
**N° (%) treatments stop due to adverse events**	8 (1.2)	0 (0)	2 (0.7)	6 (1.8)		
**N° (%) treatments stop on clinical decision**	3 (0.4)	0 (0)	1 (0.4)	2 (0.6)		
**N° (%) treatments stop for hospitalization due to COVID-19**	0 (0)	0 (0)	0 (0)	0 (0)		
**N° (%) treatments stop for patient’s decision**	59 (8.8)	0 (0)	27 (9.9)	32 (9.8)		
**N° (%) of patients hospitalized not for COVID-19 that received the treatment during follow up**	8 (1.2)	0 (0)	0 (0)	8 (2.5)	**0.007**	0.360
Median (Q1–Q3) of time-to-negative swab for SARS-CoV-2 *	12 (9–17)	13 (9–18)	11 (8–15)	13 (9–19)	**0.002**	0.605
**N° (%) of dead patients during follow up**	5 (0.7)	2 (2.9)	0 (0)	3 (0.9)	0.161	0.211

* days passed from symptoms onset, until first negative swab.

**Table 3:** Demographic and clinical data of patients included in this study grouped by the early treatment performed.

	Sotrovimab	Oral Antivirals (Molnupiravir or Nirmatrelvir/Ritonavir)	*p*
**Patients included**	**326**	**342**	**-**
**Male, N° (%)**	154 (47.2%)	174 (50.9%)	0.354
**Age in years, median (Q1–Q3)**	70 [57–80]	69 [59–78]	0.283
**Time from symptom onset to 1st drug dose, median (Q1–Q3)**	3 [2–6]	2 [1–3]	**<0.001**
**N° (%) anti-SARS CoV-2 vaccinated patients**	240 (75.0%)	310 (91.4%)	**<0.001**
**N° (%) patients who received all advised doses according to the vaccine**	179 (55.4%)	260 (79%)	**<0.001**
**N° (%) patients in chronic non-COVID-19 O_2_ therapy**	27 (8.3%)	17 (5%)	0.089
**N° (%) oncologic/hematologic active disease**	35 (10.7%)	62 (18.1%)	**0.008**
**N° (%) CKD patients**	35 (10.7%)	23 (6.7%)	0.074
**N° (%) COPD or other chronic disease patients**	44 (13.5%)	48 (14%)	0.911
**N° (%) immunocompromised, innate or acquired, patients**	71 (21.8%)	66 (19.3%)	0.444
**N° (%) obese patients**	61 (18%)	58 (17%)	0.613
**N° (%) cardiac/cerebrovascular disease patients (HF, coronary disease, cardiomyopathy, HT with related organ damage, stroke)**	159 (48.8%)	117 (34.2%)	**<0.001**
**N° (%) decompensated diabetes mellitus patients (Hb1A >/= 9.0% or 75 *mol*/*mol*) or chronic complications**	57 (17.5%)	34 (9.9%)	**0.005**
**N° (%) over 65 y/o patients**	168 (51.5%)	133 (38.9%)	**0.001**
**N° (%) liver chronic disease**	10 (3.1%)	3 (0.9%)	0.05
**N° (%) neurological disease**	27 (8.3%)	10 (3.3%)	**0.003**
**SpO2 at admission, Median (Q1–Q3)**	96 [95–98]	96 [95–97]	0.134

Abbreviations and acronyms: SARS CoV-2: Severe Acute Respiratory Syndrome Corona Virus-2; COVID-19: Corona Virus Disease-2019; CKD: Chronic Kidney Disease; COPD: Chronic Obstructive Pulmonary Disease; HF: Heart Failure; HT: Hypertension; Hb1A: Hemoglobin 1A; y/o: Years old.

**Table 4:** Outcome of patients included in this study grouped by the early treatment performed.

	Sotrovimab	Other Antivirals (Molnupiravir or Nirmatrelvir/Ritonavir)	*p*
**N° (%) of patients who finished the treatment**	283 (86.8%)	310 (90.6%)	0.433
**N° (%) treatments stopped due to adverse events**	6 (1.8%)	2 (0.7%)	
**N° (%) treatments stopped on clinical decision**	2 (0.6%)	1 (0.4%)	
**N° (%) treatments stopped for hospitalization**	0 (0%)	0 (0%)	
**N° (%) treatments stopped for patient’s decision**	32 (9.8%)	27 (9.9%)	
**N° (%) of patients hospitalized not for COVID-19 that received the treatment**	8 (2.45%)	0 (0%)	**0.003**
Median (Q1–Q3) of time-to-negative swab for SARS-CoV-2 *	13 [9–19]	11 [8–16]	**0.008**
**N° (%) of dead patients**	3 (0.9%)	2 (1.8%)	0.679

* days passed from symptoms onset until first negative swab. Abbreviations and acronyms: SARS CoV-2: Severe Acute Respiratory Syndrome Corona Virus-2; COVID-19: Corona Virus Disease-2019.

**Table 5:** Demographic and clinical data of patients included in this study grouped by time to negativization of SARS-CoV-2 specimen.

	Time to Negativization ≤ 12 Days	Time to Negativization > 12 Days	*p*
**Patients included**	**244**	**215**	**-**
**Male, N° (%)**	124 (50.8)	101 (47)	0.411
**Age in years, median (Q1–Q3)**	66 (55–76)	69 (56–80)	0.122
**Time from symptom onset to 1st drug dose, median (Q1–Q3)**	2 (1–4)	3 (2–4)	**0.045**
**N° (%) anti-SARS CoV-2 vaccinated patients**	205 (85.4)	168 (79.6)	0.104
**N° (%) patients who received all advised doses according to the vaccine**	184 (77.6)	141 (67.1)	**0.013**
**N° (%) patients in chronic non-COVID-19 O2 therapy**	16 (6.6)	12 (5.6)	0.663
**N° (%) oncologic/hematologic active disease**	35 (14.3)	26 (12.1)	0.478
**N° (%) CKD patients**	16 (6.6)	21 (9.8)	0.207
**N° (%) COPD or other chronic disease patients**	31 (12.7)	29 (13.5)	0.804
**N° (%) immunocompromised, innate or acquired, patients**	57 (23.4)	50 (23.3)	0.979
**N° (%) obese patients**	43 (17.6)	49 (22.8)	0.168
**N° (%) cardiac/cerebrovascular disease patients (HF, coronary disease, cardiomyopathy, HT with related organ damage, stroke)**	82 (33.6)	98 (45.6)	**0.009**
**N° (%) decompensated diabetes mellitus patients (Hb1A >/= 9.0% or 75 *mol*/*mol*) or chronic complications**	29 (11.9)	36 (16.7)	0.136
**N° (%) over 65 y/o patients**	97 (39.8)	92 (42.8)	0.509
**N° (%) liver chronic disease**	4 (1.6)	6 (2.8)	0.526
**N° (%) neurological disease**	11 (4.5)	10 (4.7)	0.942
**SpO_2_ at admission, Median (Q1–Q3)**	97 (95–98)	96 (95–98)	0.146
**N° (%) early treatment performed** - **Molnupiravir** - **Nirmatrelvir/r** - **Sotrovimab**	30 (12.3) 111 (45.5) 103 (42.2)	31 (14.4) 110 (51.2) 74 (34.4)	0.054
**N° (%) early treatment performed** - **Oral antiviral** - **Sotrovimab**	141 (57.8) 103 (42.2)	105 (48.8) 110 (51.2)	0.055

Abbreviations and acronyms: SARS CoV-2: Severe Acute Respiratory Syndrome Corona Virus-2; COVID-19: Corona Virus Disease-2019; CKD: Chronic Kidney Disease; COPD: Chronic Obstructive Pulmonary Disease; HF: Heart Failure; HT: Hypertension; Hb1A: Hemoglobin 1A; y/o: Years old.

**Table 6:** Multivariable logistic regression investigating the predictors associated with a time to negativization (days passed from symptoms onset until first negative swab) greater than twelve days.

	*p*	OR	IC 95%
**Time from symptom onset to 1st drug dose**	0.812	1.010	0.930–1.097
**Early treatment performed** - Sotrovimab[Ref] - Oral antiviral	0.416	1.184	0.789–1.777
**patients who received all advised doses according to the vaccine**	0.096	0.686	0.440–1.069
**N° (%) cardiac/cerebrovascular disease patients (HF, coronary disease, cardiomyopathy, HT with related organ damage, stroke)**	**0.021**	**1.585**	**1.072–2.345**

**Table 7:** Demographic and clinical data of patients dead or alive at the end of follow up.

	Alive at the End of Follow Up	Dead During Follow Up	*p*
**Patients included**	**663**	**5**	
**Male, N° (%)**	327 (49.3)	1 (20)	0.374
**Age in years, median (Q1–Q3)**	69 (58–79)	70 (39–82)	0.792
**Time from symptom onset to 1st drug dose, median (Q1–Q3)**	2 (1–4)	2 (1–5)	0.659
**N° (%) anti-SARS CoV-2 vaccinated patients**	546 (83.5)	4 (80)	0.596
**N° (%) patients who received all advised doses according to the vaccine**	436 (67.4)	3 (60)	0.664
**N° (%) patients in chronic non-COVID-19 O_2_ therapy**	42 (6.3)	2 (40)	**0.037**
**N° (%) oncologic/hematologic active disease**	97 (14.6)	0 (0)	1
**N° (%) CKD patients**	57 (8.6)	1 (20)	0.366
**N° (%) COPD or other chronic disease patients**	90 (13.6)	2 (40)	0.142
**N° (%) immunocompromised, innate or acquired, patients**	133 (20.1)	4 (80)	0.07
**N° (%) obese patients**	118 (17.8)	1 (20)	1
**N° (%) cardiac/cerebrovascular disease patients (HF, coronary disease, cardiomyopathy, HT with related organ damage, stroke)**	273 (41.2)	3 (60)	0.409
**N° (%) decompensated diabetes mellitus patients (Hb1A >/= 9.0% or 75 *mol*/*mol*) or chronic complications**	89 (13.4)	2 (40)	0.139
**N° (%) over 65 y/o patients**	299 (45.1)	2 (40)	1
**N° (%) liver chronic disease**	13 (2)	0 (0)	1
**N° (%) neurological disease**	37 (5.6)	0 (0)	1
**SpO_2_ at admission, Median (Q1–Q3)**	96 (95–98)	92 (92–95)	**0.005**
**N° (%) early treatment performed** - **Molnupiravir** - **Nirmatrelvir/r** - **Sotrovimab**	67 (10.1) 273 (41.2) 323 (48.7)	2 (40) 0 (0) 3 (60)	**0.039**
**N° (%) early treatment performed** - **Oral antiviral** - **Sotrovimab**	340 (51.3) 323 (48.7)	2 (40) 3 (60)	0.679

Abbreviations and acronyms: SARS CoV-2: Severe Acute Respiratory Syndrome Corona Virus-2; COVID-19: Corona Virus Disease-2019; CKD: Chronic Kidney Disease; COPD: Chronic Obstructive Pulmonary Disease; HF: Heart Failure; HT: Hypertension; Hb1A: Hemoglobin 1A; y/o: Years old.

**Table 8:** Multivariable logistic regression investigating the predictors of death.

	*p*	OR	IC 95%
**Immunocompromised, innate or acquired, patients**	**0.013**	16.929	1.835–156.170
**Patients in chronic non-COVID-19 O_2_ therapy**	**0.014**	10.714	1.623–70.725
**Early treatment performed** - **Sotrovimab [Ref]** - **Oral antiviral**	0.755	1.342	0.211–8.530

The sub-analysis performed considering patients dead and alive at the end of follow up showed that the most important variables related to death at follow up were the presence at baseline of an innate or acquired immunocompromising disease (OR 16.974, 95% CI 1.836–156.949, *p* = 0.013) and chronic non-COVID-19 O_2_ therapy (OR 11.292, 95% CI 1.698–75.119, *p* = 0.012); no correlation was found between early anti-SARS-CoV-2 treatment and death.

## Data Availability

The data will be made available by the authors on request.

## Supplementary Materials

The following supporting information can be downloaded at https://www.mdpi.com/article/10.3390/pathogens14030216/s1: Supplementary Table S1: Demographic and clinical data of patients included in the study grouped by the early treatment performed, nirmatrelvir/r or sotrovimab; Supplementary Table S2: Outcome of patients included in the study grouped by the early treatment performed, molnupiravir or sotrovimab; Supplementary Table S3: Demographic and clinical data of patients included in the study grouped by the early treatment performed, nirmatrelvir/r or sotrovimab; Supplementary Table S4: Outcome of patients included in the study grouped by the early treatment performed, molnupiravir or sotrovimab; Supplementary Table S5: Demographic and clinical data of patients included in the study grouped by the hospitalization during follow up for non-COVID-19 conditions; Supplementary Table S6: Outcome data of patients included in the study grouped by the hospitalization during follow up for non-COVID-19 conditions; Supplementary Table S7: Cox regression univariate analysis for time to negativization.
