# Supplementary material for: Efficacy and Safety of Sotrovimab Versus Oral Antiviral for Early Treatment in High-Risk Patients in Omicron Era: A Multicenter Retrospective Study
Source: Pathogens. 2025 Feb 22;14(3):216. doi: 10.3390/pathogens14030216 (PMC11945712; doi:10.3390/pathogens14030216)
Supplement: Supplementary file 1 [file pathogens-14-00216-s001.zip › pathogens-3432666-supplementary.pdf]

Supplementary Materials

Supplementary Table 1: Demographic and clinical data of patients included in the study grouped by the early treatment performed, nirmatrelvir/r or sotrovimab

	Nirmatrelvir/r	Sotrovimab	p
**Patients included**	**273**	**326**	
**Males, N°(%)**	139(50.9)	154(47.2)	0.412
**Age in years, median (Q1-Q3)**	70(59-78)	70(57-80)	0.929
**Time from symptom onset to 1st drug dose, median (Q1-Q3)**	2(1-3)	3(2-6)	**0.0001**
**N° (%) anti-SARS CoV-2 Vaccinated patients**	255(94.1)	240(75)	**0.0001**
**N°(%) patients who received all advised doses according to the vaccine**	210(80.5)	179(55.4)	**0.0001**
**N°(%) patients in chronic non COVID-19 O2 therapy**	9(3.3)	27(8.3)	**0.015**
**N°(%) oncologic/hematologic active disease**	52(19)	35(10.7)	**0.005**
**N°(%) CKD patients**	21(7.7)	35(10.7)	0.209
**N°(%) COPD or other chronic disease patients**	39(14.3)	44(13.5)	0.813
**N°(%) Immunocompromised, innate or acquired, patients**	46(16.8)	71(21.8)	0.148
**N°(%) obese patients**	40(14.7)	61(18.7)	0.191
**N°(%) cardiac/cerebrovascular disease patients (HF, coronary disease, cardiomyopathy, HT with related organ damage, stroke)**	79(28.9)	159(48.8)	**0.0001**
**N°(%) decompensated diabetes mellitus patietns (Hb1A>/= 9,0% or 75mol/mol) or chronic complications**	20(7.3)	57(17.5)	**0.0001**
**N°(%) over 65 y/o patients**	107(39.2)	168(51.5)	**0.003**
**N°(%) Liver chronic disease**	2(0.7)	10(3.1)	**0.037**
**N°(%) Neurological disease**	8(2.9)	27(8.3)	**0.005**
**SpO2 at admission, Median (Q1-Q3)**	96(95-97)	96(95-98)	0.067

Supplementary Table 2: Outcome of patients included in the study grouped by the early treatment performed, molnupiravir or sotrovimab

	**Nirmatrelvir/r**	**Sotrovimab**	**p**
**N°(%) of patients who finished the treatment**	243(89)	283(86.8)	0.387
**N°(%) treatments stop due to adverse events**	2(0.7)	6(1.8)	
**N°(%) treatments stop on clinical decision**	1(0.4)	2(0.6)	
**N°(%) treatments stop for hospitalization due to COVID-19**	0(0)	0(0)	
**N°(%) treatments stop for patient's decision**	27(9.9)	32(9.8)	
**N°(%) of patients hospitalized not for COVID-19 that received the treatment**	0(0)	8(2.5)	**0.007**
**Median (Q1-Q3) of Time-to-negative swab for SARS-CoV-2**	11(8-15)	13(9-19)	**0.002**
**N°(%) of dead patients**	0(0)	3(0.9)	0.161

Supplementary Table 3: Demographic and clinical data of patients included in the study grouped by the early treatment performed, nirmatrelvir/r or sotrovimab

	**Molnupiravir**	**Sotrovimab**	**p**
**Patients included**	**69**	**326**	
**Males, N°(%)**	35(50.7)	154(47.2)	0.691
**Age in years, median (Q1-Q3)**	63(57-78)	70(57-80)	0.235
**Time from symptom onset to 1st drug dose, median (Q1-Q3)**	3(2-4)	3(2-6)	**0.015**
**N° (%) anti-SARS CoV-2 Vaccinated patients**	55(80.9)	240(75)	0.302
**N°(%) patients who received all advised doses according to the vaccine**	50(73.5)	179(55.4)	**0.007**
**N°(%) patients in chronic non COVID-19 O2 therapy**	8(11.6)	27(8.3)	0.379
**N°(%) oncologic/hematologic active disease**	10(14.5)	35(10.7)	0.372
**N°(%) CKD patients**	2(2.9)	35(10.7)	**0.041**
**N°(%) COPD or other chronic disease patients**	9(13)	44(13.5)	0.920
**N°(%) Immunocompromised, innate or acquired, patients**	20(29)	71(21.8)	0.197
**N°(%) obese patients**	18(26.1)	61(18.7)	0.164
**N°(%) cardiac/cerebrovascular disease patients (HF, coronary disease, cardiomyopathy, HT with related organ damage, stroke)**	38(55.1)	159(48.8)	0.342
**N°(%) decompensated diabetes mellitus patietns (Hb1A>/= 9,0% or 75mol/mol) or chronic complications**	14(20.3)	57(17.5)	0.581
**N°(%) over 65 y/o patients**	26(37.7)	168(51.5)	**0.037**
**N°(%) Liver chronic disease**	1(1.4)	10(3.1)	**0.697**
**N°(%) Neurological disease**	2(2.9)	27(8.3)	0.200
**SpO2 at admission, Median (Q1-Q3)**	97(95-98)	96(95-98)	0.395

Supplementary Table 4: Outcome of patients included in the study grouped by the early treatment performed, molnupiravir or sotrovimab

	Molnupiravir	Sotrovimab	p
**N°(%) of patients who finished the treatment**	67(97.1)	283(86.8)	**0.028**
**N°(%) treatments stop due to adverse events**	0(0)	6(1.8)	
**N°(%) treatments stop on clinical decision**	0(0)	2(0.6)	
**N°(%) treatments stop for hospitalization due to COVID-19**	0(0)	0(0)	
**N°(%) treatments stop for patient's decision**	0(0)	32(9.8)	
**N°(%) of patients hospitalized not for COVID-19 that received the treatment**	0(0)	8(2.5)	0.360
**Median (Q1-Q3) of Time-to-negative swab for SARS-CoV-2**	13(9-18)	13(9-19)	0.605
**N°(%) of dead patients**	2(2.9)	3(0.9)	0.211

Supplementary Table 5:Demographic and clinical data of patients included in the study grouped by the hospitalization during follow up for non-COVID-19 conditions.

	Patients not hospitalized during Follow up	Patients hospitalized not for COVID-19	p
**Patients included**	**8**	**660**	**-**
**Males, N°(%)**	326(50.6)	2(25)	0.287
**Age in years, median (Q1-Q3)**	70(59-80)	63(41-76)	0.260
**Time from symptom onset to 1st drug dose, median (Q1-Q3)**	2(1-4)	5(1-5)	0.558
**N° (%) anti-SARS CoV-2 Vaccinated patients**	545(83.7)	5(62.5)	0.131
**N°(%) patients who received all advised doses according to the vaccine**	437(67.9)	2(25)	**0.017**
**N°(%) patients in chronic non COVID-19 O2 therapy**	44(6.7)	0(0)	1.0
**N°(%) oncologic/hematologic active disease**	96(14.5)	1(12.5)	1.0
**N°(%) CKD patients**	57(8.6)	1(12.5)	0.518
**N°(%) COPD or other chronic disease patients**	92(13.9)	0(0)	0.607
**N°(%) Immunocompromised, innate or acquired, patients**	137(20.8)	0(0)	0.371
**N°(%) obese patients**	116(17.6)	3(37.5)	0.156
**N°(%) cardiac/cerebrovascular disease patients (HF, coronary disease, cardiomyopathy, HT with related organ damage, stroke)**	270(40.9)	6(75)	0.071
**N°(%) decompensated diabetes mellitus patietns (Hb1A>/= 9,0% or 75mol/mol) or chronic complications**	89(13.5)	2(25)	0.299
**N°(%) over 65 y/o patients**	298(45.2)	3(37.5)	0.736
**N°(%) Liver chronic disease**	13(2)	0(0)	1.0
**N°(%) Neurological disease**	37(5.6)	0(0)	1.0

**SpO2 at admission, Median (Q1-Q3)**	96(95-98)	95(94-98)	0.231
**N°(%) who performed treatment with sotrovimab**	318(48.2)	8(100)	**0.03**

Abbreviations and acronyms: SARS CoV-2: Severe Acute Respiratory Syndrome Corona Virus-2; COVID-19: Corona Virus Disease-2019; CKD: Chronic Kidney Disease; COPD: Chronic Obstruc-tive Pulmonary Disease; HF: Heart Failure; HT: Hypertension; Hb1A: Hemoglobin 1A; y/o: Years old.

Supplementary Table 6: Outcome data of patients included in the study grouped by the hospitalization during follow up for non-COVID-19 conditions.

	**Patients hospitalized not for COVID-19**	**Patients not hospitalized during Follow up**	**p**
**N°(%) of patients who finished the treatment**	591(89.5)	2(25)	**<0.001**
**N°(%) treatments stop due to adverse events**	5(0.8)	0(0)	
**N°(%) treatments stop on clinical decision**	**2(0.3)**	**6(75)**	
**N°(%) treatments stop for hospitalization due to COVID-19**	3(0.5)	0(0)	
**N°(%) treatments stop for patient's decision**	59(8.9)	0(0)	
**Median (Q1-Q3) of Time-to-negative swab for SARS-CoV-2 ***	12(9-17)	13(1-17)	0.499
**N°(%) of dead patients**	5(0.8)	0(0)	1.0

**days passed from symptoms onset, until first negative swab*

Supplementary Table 7: Cox regression univariate analysis for time to negativization.

	p	Hazard ratio	95%CI
Males	0.989	0.998	0.830-1.200
Anti-SARS CoV-2 vaccinated patients	0.108	1.223	0.957-1.562
Patients who received all advised doses according to the vaccine	0.090	1.198	0.972-1.477
Patients in chronic non COVID-19 O2 therapy	0.364	0.836	0.568-1.231
oncologic/hematologic active disease	0.628	0.935	0.711-1.228
CKD patients	0.232	0.814	0.581-1.141
COPD or other chronic disease patients	0.372	0.883	0.671-1.161
Immunocompromised, innate or acquired, patients	0.413	0.913	0.735-1.135
Obese patients	0.246	0.873	0.694-1.098
Cardiac/cerebrovascular disease patients (HF, coronary disease, cardiomyopathy, HT with related organ damage, stroke)	0.110	0.858	0.711-1.036
Decompensated diabetes mellitus patietns (Hb1A>/= 9,0% or 75mol/mol) or chronic complications	0.164	0.829	0.637-1.079
Over 65 y/o patients	0.268	0.900	0.746-1.085
Liver chronic disease	0.795	0.920	0.492-1.723
Neurological disease	0.408	1.204	0.776-1.868
Patients who performed treatment with oral antiviral	**0.019**	**0.801**	**0.666-0.964**
